# Crystal Structure of a GH3 β-Glucosidase from the Thermophilic Fungus *Chaetomium thermophilum*

**DOI:** 10.3390/ijms20235962

**Published:** 2019-11-27

**Authors:** Imran Mohsin, Nirmal Poudel, Duo-Chuan Li, Anastassios C. Papageorgiou

**Affiliations:** 1 Turku Bioscience, University of Turku and Åbo Akademi University, 20520 Turku, Finland; dr.mohsinimran@hotmail.com (I.M.); nirpou@utu.fi (N.P.); 2 Department of Mycology, Shandong Agricultural University, Taian 271018, China

**Keywords:** glycoside hydrolase, cellulose degradation, thermophilic fungus, β-glucosidases, *Chaetomium thermophilum*, protein structure, fungal enzymes

## Abstract

Beta-glucosidases (β-glucosidases) have attracted considerable attention in recent years for use in various biotechnological applications. They are also essential enzymes for lignocellulose degradation in biofuel production. However, cost-effective biomass conversion requires the use of highly efficient enzymes. Thus, the search for new enzymes as better alternatives of the currently available enzyme preparations is highly important. Thermophilic fungi are nowadays considered as a promising source of enzymes with improved stability. Here, the crystal structure of a family GH3 β-glucosidase from the thermophilic fungus *Chaetomium thermophilum* (*Ct*BGL) was determined at a resolution of 2.99 Å. The structure showed the three-domain architecture found in other β-glucosidases with variations in loops and linker regions. The active site catalytic residues in *Ct*BGL were identified as Asp287 (nucleophile) and Glu517 (acid/base). Structural comparison of *Ct*BGL with Protein Data Bank (PDB)-deposited structures revealed variations among glycosylated Asn residues. The enzyme displayed moderate glycosylation compared to other GH3 family β-glucosidases with similar structure. A new glycosylation site at position Asn504 was identified in *Ct*BGL. Moreover, comparison with respect to several thermostability parameters suggested that glycosylation and charged residues involved in electrostatic interactions may contribute to the stability of the enzyme at elevated temperatures. The reported *Ct*BGL structure provides additional insights into the family GH3 enzymes and could offer new ideas for further improvements in β-glucosidases for more efficient use in biotechnological applications regarding cellulose degradation.

## 1. Introduction

Beta-glucosidases (β-glucosidases) are key cellulolytic enzymes that catalyze the hydrolysis of cellobiose to glucose. They are terminal enzymes of the cellulase system as they act during the final step of the cellulose degradation. Although beta-glucosidases do not act directly on cellulose, their activity is important to circumvent the inhibitory effect of cellobiose on endoglucanases and cellobiohydrolases. Consequently, inclusion of β-glucosidases in cellulase preparations can synergistically increase the hydrolytic efficiency of other cellulolytic enzymes [1].

Beta-glucosidases (β-glucoside glucohydrolases, EC 3.2.1.21; BGL) have been found in the glycoside hydrolase (GH) families GH1, GH3, GH5, GH9, GH30, and GH116 of the CAZy database [2]. They are anomeric configuration-retaining enzymes that operate through the canonical double-displacement glycosidase mechanism, except those of the GH9 family.

Despite their extensive use in biotechnological application, structural data on fungal β-glucosidases are still scarce. Many fungal BGLs are classified as part of the GH3 family, one of the largest CAZy families with over 13,600 annotated protein sequences. Two fungal β-glucosidase structures currently available are produced by the mesophilic fungus *Aspergilus aculeatus* [3] and the moderate thermophilic *Rasamsonia emersonii* [4], respectively. A third structure produced by the mesophilic fungus *Trichoderma reesei* of BGL1 has been deposited in the Protein Data Bank (PDB), but no paper has been published yet. Recently, the structure of the filamentous fungus *Neurospora crassa* Cel3A (*Nc*Cel3A) was reported at 2.25 Å resolution [5], as well as structures of GH3 β-glucosidases from *Hypocrea jecorina* (a teleomorph of *Trichoderma reesei*) [6], *Aspergilus niger* [7], *Aspergillus fumigatus,* and *Aspergillus oryzae* [8].

The first structure of a β-glucosidase, that of barley (*Hordeum vulgare*) exo-1,3-1,4-glucanase ExoI (*Hv*ExoI) [9] showed a two-domain architecture: An N-terminal (α/β)8 TIM-barrel that hosts the catalytic nucleophile aspartate and an (α/β)_6_ sandwich domain where the acid/base glutamate resides. Subsequent studies, however, revealed variations in the modular architecture of GH3 β-glucosidases with the first domain to adopt a collapsed/incomplete triosephosphate isomerase (TIM)-barrel domain and the presence of a third domain with a fibronectin FnIII-like fold. The function of the third domain is still unknown, although it has been suggested to stabilize the incomplete TIM-barrel domain [4]. Notably, a fourth domain has also been identified in *Kluyveromyces marxianus* and *Pseudoalteromonas* sp. BB1 β-glucosidases [10,11].

Thermophilic fungi have attracted considerable attention in recent years as an alternative reservoir of thermostable cellulases for cellulose degradation [12,13]. Importantly, thermophilic fungi can produce thermostable enzymes that can be used at temperatures up to 70 °C, whereas enzymes from mesophilic organisms are typically active up to 50 °C. The importance of using thermophilic cellulases in cellulose degradation stems from the fact that, at higher temperatures, cellulose swells and becomes more susceptible to breaking. Various thermophilic fungi have been studied in recent years and their β-glucosidases have been characterized [12]. Understanding the structure–function–stability relationships in fungal β-glucosidases is therefore important for finding new and better alternatives for industrial biocatalysts. Hydrolysis rate, inhibitors, and stability are considered critical factors for the efficient use of β-glucosidases in complex biomass hydrolysis [14]. Moreover, β-glucosidases have been suggested for use in the synthesis of various glycoconjugates, and a GH3 β-glucosidase from the thermophilic fungus *Myceliophthora thermophila* has been found to act as an efficient biocatalyst in alkyl glycoside synthesis [15].

Here, we report the crystal structure of a β-glucosidase from the thermophilic filamentous fungus *Chaetomium thermophilum* (*Ct*BGL) at a resolution of 2.99 Å, and compare it with other β-glucosidases of the GH3 family. This is the second structure of a β-glucosidase produced by a thermophilic fungus, and therefore, it is expected to provide further insights into the structure–function relationship of this family of enzymes in high-temperature settings.

## 2. Results

### 2.1. Quality of the Structure

The crystal structure of *Ct*BGL was refined using data up to 2.99 Å resolution. The final *R*_cryst_ and *R*_free_ (5% of the reflections excluded from refinement) were 0.201 and 0.252, respectively (Table 1). The structure exhibits good stereochemistry despite the limited resolution with root mean square deviation (rmsd) in bond lengths and bond angles of 0.007 Å and 0.94°, respectively. The Ramachandran plot shows 92.8% of the residues in the most favorable regions and 1.0% in disallowed regions. Residues that fall into the disallowed regions belong to flexible parts of the structure with weak electron density. Structure quality statistics for *Ct*BGL fall within the distribution found in other crystal structures of similar resolution as analyzed and displayed by POLYGON [15]. The refined structure contains 836 residues of the mature protein, starting at residue Trp32. The full-length cDNA encodes for a 867-residue enzyme and the first 16 residues have been suggested to act as a secretion signal peptide [16]. Residues 17–31 at the N-terminal were not modeled, owing to lack of adequate electron density, possibly due to high flexibility. The enzyme crystallized with one molecule in the crystallographic asymmetric unit in contrast to other β-glucosidases that crystallize either with two molecules such as the β-glucosidases from *Aspergillus oryzae* (*Aoβ*G) and *A. fumigatus* (*Afβ*G) [8], *A. aculeatus* (*Aaβ*G) [8] and *Nc*Cel3A [5], or with four molecules in the asymmetric unit such as *Re*Cel3A [4]. The solvent content is unusually high (~77%) for a protein crystal, which could explain the low resolution of the data and the disintegration of the crystals during manipulation prior to mounting. The NCS dimer found in other β-glucosidases is seen in *Ct*BGL as a crystallographic dimer that involves the −Y, −X, −Z+ ½ symmetry-related molecule.

### 2.2. Overall Structure Comparisons

**Figure 1:**
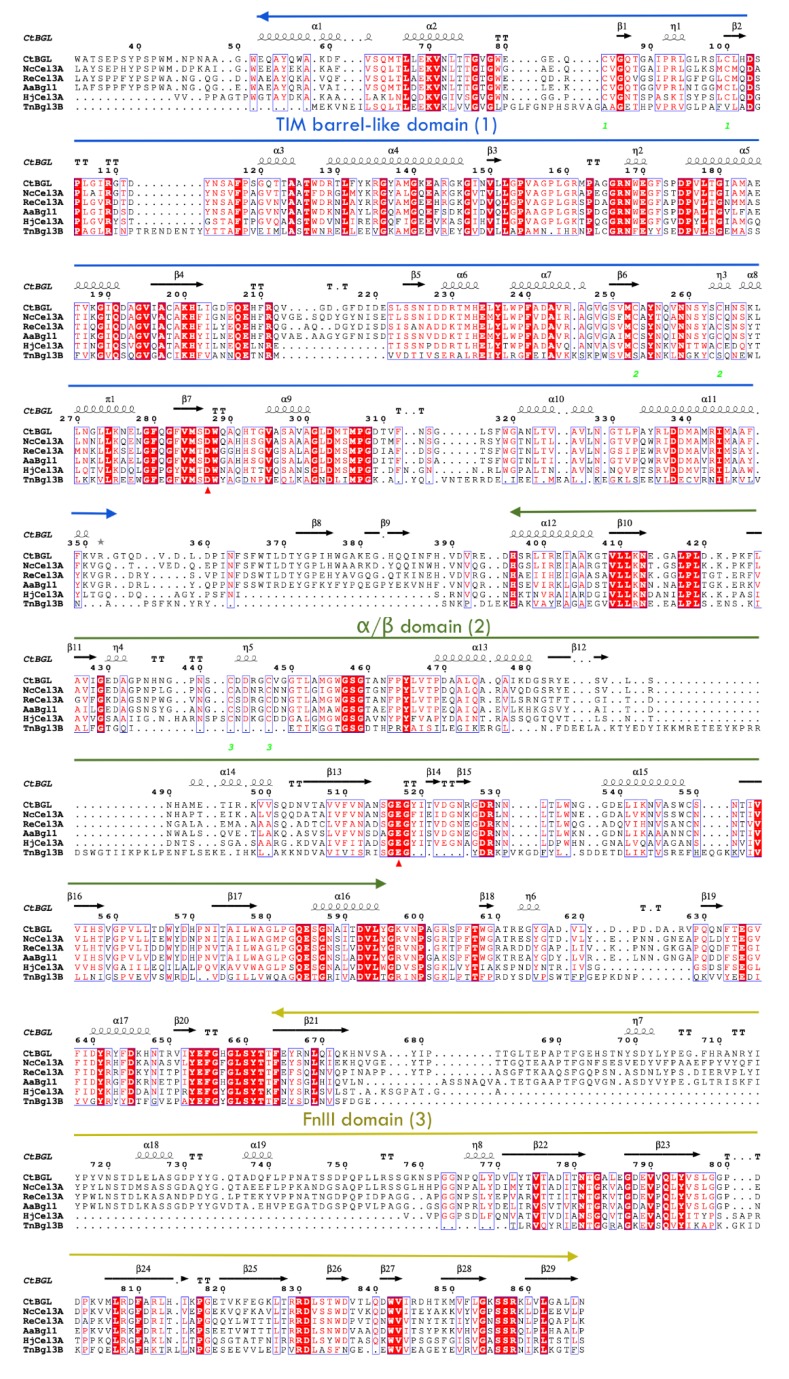
Structure-based sequence alignment of *Ct*BGL with members of GH3 family. Secondary structure elements are shown on the top of the alignment, while the red triangles below the alignment indicate the catalytic conserved residues (Asp287 and Glu517 in *Ct*BGL). Disulfide bonds are depicted with green numbers. The three domains are indicated. A column is framed if more than 70% of its residues are similar according to physicochemical properties. Frames in red background with white letters depict strict identity. The figure was constructed with ESPript [16].

Structure-based sequence alignment (Figure 1) showed that *Ct*BGL has the highest structural similarity with *Nc*Cel3A (PDB id 5nbs), as indicated by the root mean square deviation (rmsd) of 0.60 Å for 831 aligned residues (sequence identity 72%), followed by *Aspergilus fumigatus Afβ*G (PDB id 5fji; rmsd 0.81 Å; seq. identity 62%), *Re*Cel3A (PDB id 5ju6; rmsd 0.84 Å; 62.0%) and *Aspergillus aculeatus Aa*Bgl1 (4iig; 0.83 Å; 62.0%). Lower similarities were found with *Hypocrea jecorina Hj*Cel3A (3zyz; 1.21 Å; 47%).

### 2.3. Description of the Structure

**Figure 2:**
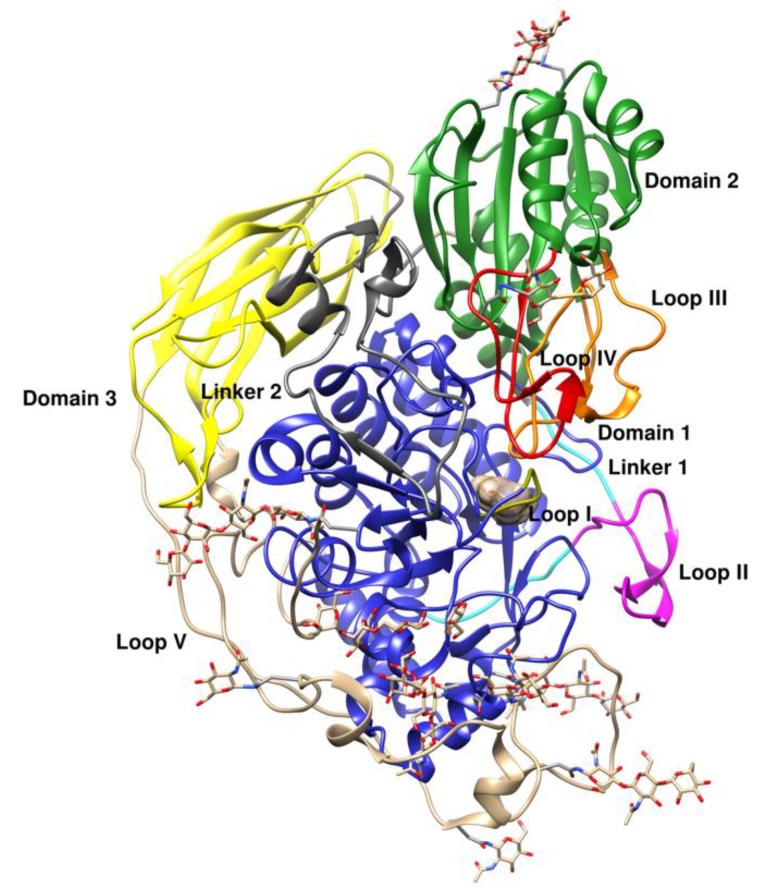
Overall crystal structure of *Ct*BGL in ribbon representation. The domains, linkers, and loops are shown in different colors and labeled: Catalytic TIM barrel-like domain (blue), α/β sandwich domain (green), FnIII domain (yellow), loop I (gold), linker 1 (cyan), loop II or insertion region (magenta), loop III (orange), loop IV (orange–red), linker 2 (gray), loop V (brown). The N-glycans and glucose in the active site are shown as gray sticks. The figure was created with UCSF Chimera [17].

The structure of *Ct*BGL (Figure 2) consists of three distinct domains, similar to other GH3 family members: A catalytic triose phosphate isomerase (TIM) barrel-like domain (Leu51–Ser354), an α/β sandwich domain (His396–Gly596) and a FnIII (fibronectin type III) domain (Thr663–Gln867) with a prominent insertion region (677–766). A linker region (residues 355–394) connects domain 1 to domain 2 and a second linker region (600–662) connects domain 2 to domain 3. The 39-residue linker between domain 1 and domain 2 is comparable to that in *Nc*Cel3A and significantly longer than the linker in other GH3 β-glucosidases such as *Hj*Cel3A and *Hv*ExoI with only 18 and 16 residues in length, respectively. In *Nc*Cel3A, the linker comprises 42 residues (residues 341–383) with a 25-residue (residues 351–376) insertion previously described as a hydrophobic linker responsible for the activation of *Re*Cel3A and *Aa*Bgl in organic solvents [5]. This insertion has been coined as loop II and contains several aromatic residues, of which *Ct*GBL Phe364 (Phe352 in *Nc*Cel3A) and *Ct*GBL Trp367 (Trp355 in *Nc*Cel3A) are the most conserved. Moreover, loop II contains residues Phe366, Trp367, and Trp377 that line up one side of the substrate-binding site, and they are also found in *Nc*Cel3A (Phe354, Trp355, and Trp365, respectively) with variations in other β-glucosidases, such as *Re*Cel3A and *Aa*Bgl1. It has been suggested that this loop is stabilized by interactions with the N-glycans from a neighboring glycosylated Asn residue (Asn57 in *Nc*Cel3A) [5]. In *Ct*BGL, the equivalent residue is Asn72, which is also glycosylated (see below).

**Figure 3:**
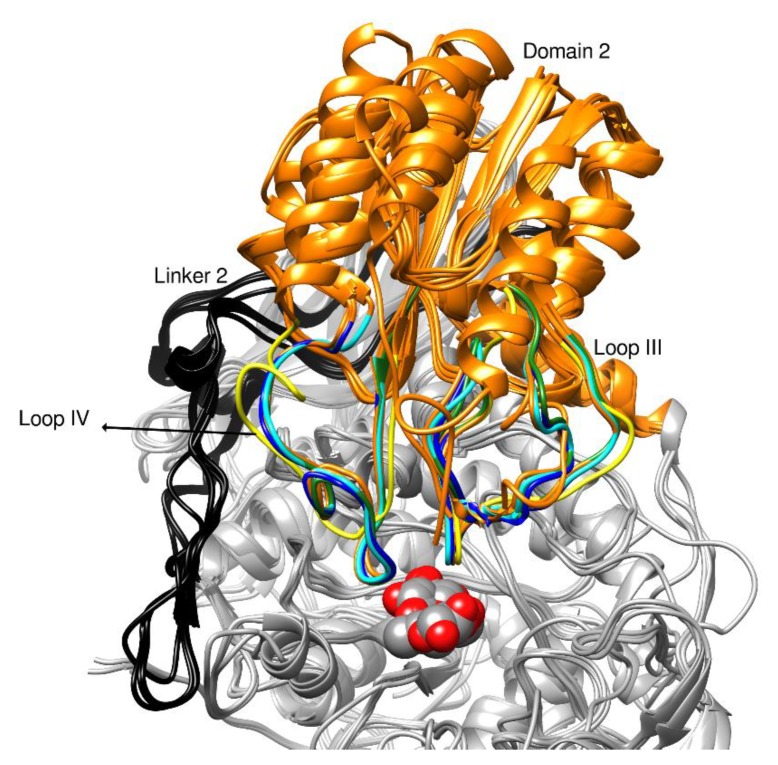
Overview of domain 2 in ribbon representation *Ct*BGL superimposed with the compared structures. Domain 2 is colored orange; loops III and IV in *Ct*BGL, *Nc*Cel3A, *Re*Cel3A, and *Tn*Bgl3B are colored in green, blue, cyan, and yellow, respectively, for structural comparisons. Linker 2 loop is colored in black, and the active site is depicted by the bound BGC in sphere representation.

*Ct*BGL second domain, an (α/β)_6_ sandwich fold, is structurally well conserved among all the GH3 enzymes. It consists of residues His396 to Gly596 and includes loops III and IV that encompass residues Gly433–Val467 and Ala513–Asn536, respectively (Figure 3). Loop III houses the conserved cysteine residues Cys442 and Cys447, which form a disulfide bridge that stabilizes the folded loop III architecture. Ser458 and Asp444 are also highly conserved in all compared structures. This domain hosts the catalytic acid Glu517 in loop IV, which is found to be conserved in all of the compared β-glucosidases, *Ct*BGL, *Nc*Cel3A, *Re*Cel3A, *Hj*Cel3A, *Aa*Bgl1, and *Thermotoga neapolitana* β-glucosidase 3B (*Tn*Bgl3B). Moreover, the architecture and location of loops III and IV, which constitute one site of the active site cavity in between loops I and II, are also found conserved. Ser458, Asp444, and Glu517 are found pointing downwards from the loop regions and towards the active site.

Domain 2 of the *Ct*BGL structure is followed by a second linker region (Linker 2; residues Lys597–Ser660). This linker region exhibits an extended structure, which almost resembles a boundary that separates domain I and domain II from domain III. The extended linker region probably plays a role in stabilizing loops I and IV.

**Figure 4:**
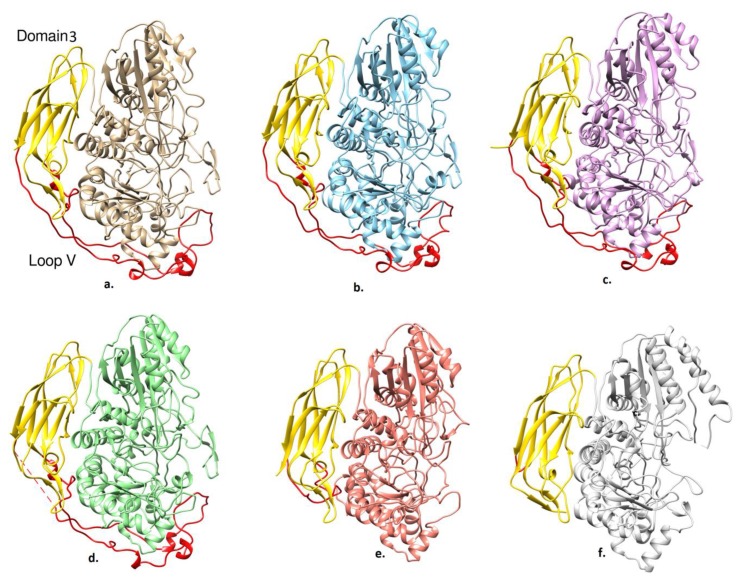
Loop V comparisons in (**a**) *Ct*BGL, (**b**) *Nc*Cel3A, (**c**) *Re*Cel3A, (**d**) AaBgl1, (**e**) *Hj*Cel3A, and (**f**) *Tn*Bgl3B. Domain 3 is colored in yellow and the loop V is represented by red coloration. Domains 1 and 2 are colored in the same color in each enzyme and differently amongst the enzymes.

The FnIII-like domain 3 was first observed in the GH3 family in the *Tn*Bgl3B structure [18]. It consists of residues Tyr661–Asn867 that form a beta sandwich composed of a total of nine β-strands arranged in two layers of β-sheets with three and four β-strands, respectively. Loop V takes an extended structure and encompasses domain I. Importantly, loop V is present in *Nc*Cel3A, *Re*Cel3A, and *Aa*Bgl1, but it is absent in *Tn*Bgl3B and *Hj*Cel3A (Figure 4). Several conserved aromatic residues, namely, Tyr716, Tyr718, Tyr733, and Phe740, are found in loop V. Tyr716 and Tyr733 are found to be conserved in all structures except *Hj*Cel3A and *Tn*Bgl3B, where loop V is absent, while Tyr718 is replaced by Trp in *Re*Cel3A and *Aa*Bgl1. Moreover, Phe740 is replaced by a Tyr residue in *Re*Cel3A and a His residue in *Aa*Bgl1. These conserved aromatic residues form π-stack interactions with *N-*acetyl-*β*-d-glucosamine (GlcNAc) residues. Also, conserved in loop V is Asn720, which was found to be N-glycosylated.

### 2.4. Glycosylation Sites

**Figure 5:**
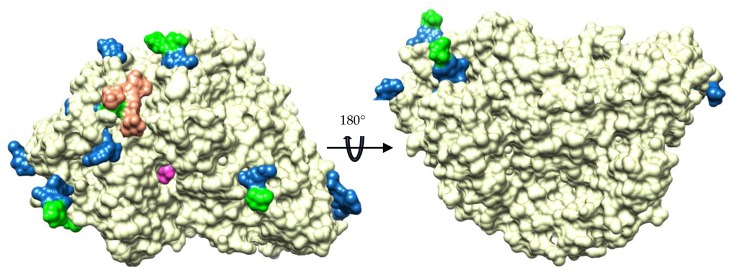
Distribution of glycosylation sites in the *Ct*BGL structure. GlcNAc is shown in blue, *β*-d-mannose in green, α-d-mannose in orange, and BGC in magenta.

Potential glycosylation sites were observed in the *Ct*BGL structure in 10 positions, all in Asn residues (Table 2). The glycosylation sites found in *Ct*BGL when compared with other structures showed some variations. A unique glycosylation site was observed in *Ct*BGL at Asn504 on the surface of the molecule, where the corresponding aligned structures had either a different residue or a gap in the structure-based alignment (Table 3). Asn259 was found highly conserved and glycosylated in all structures. Moreover, *Re*Cel3A possess 16 glycosylation sites [4], similarly to *Aspergillus* β-glucosidases [8]. On the other hand, *Hj*Cel3A showed the lowest number of glycosylation sites, with only two glycosylated sites (Asn208 and Asn310 in chains A and B, respectively). As observed before in this class of β-glucosidases, all glycosylation sites in *Ct*BGL were also located on one face of the molecule (Figure 5).

A total of 27 glycan moieties were found in *Ct*BGL. The glycans ranged in length from single GlcNAc to longer chain. The longest glycosylation chain was composed of eight residues. The overall degree of glycosylation in *Ct*BGL can be considered as moderate when compared against other glycosylation chains, such as those found in *Re*Cel3A and *Aa*BGL1, with the longest chain composed of 10 residues and 45–50 glycosylation residues in total per chain in the crystallographic asymmetric unit. Interestingly, the glycosylation pattern in *Ct*BGL showed high GlcNAc-type N-glycans, while *Re*Cel3A and *Aa*BGL1 displayed high mannose-type N-glycans. A total of 15 GlcNAc residues were identified in *Ct*BGL. Out of the total 10 glycosylation sites in *Ct*BGL, three of them were found to consist of a single GlcNAc monosaccharide. Single GlcNAc monosaccharides in other enzymes, such as *Aa*BGL1, were obtained possibly as a result of a treatment with endoglycosidase H prior to crystallization. In the case of *Ct*BGL, no endoglycosidase treatment was employed. Similarly, single GlcNAc molecules were found in *Afβ*G (in Asn543 and Asn715) without any attempt of enzymatic cleavage. It is possible that, in these cases, the enzymes were subjected to endoglycosidase activity during the expression stage. The other seven N-glycosylation sites in *Ct*BGL ranged from two to eight monosaccharides. The N-glycan at *Ct*BGL Asn329 was located in domain I of the enzyme and consisted of eight monosaccharides (two GlcNAc, five α-d-Man, and one *β*-d-Man). Extra densities that could accommodate another two monosaccharide molecules were present in the electron-density map, but they were not modeled owing to lack of clarity. It was, nevertheless, the largest N-glycan structure in *Ct*BGL and was involved in forming multiple H-bonds with the enzyme residues at Thr293, Asp730, Val295, Gly734, and Tyr733 and a pi-sigma interaction with Tyr733 residue. The structural equivalent Asn residue in *Aa*Bgl1 (Asn322) had a long-length glycan with eight Man and two GlcNAc that were also involved in extensive stabilizing interactions with domain I. N-glycans at positions Asn72 and Asn720 were located in domains I and III, respectively, with loop V residues in between them. They both participated in interactions with protein residues. The N-glycan at Asn72 was found to interact in conventional H-bonding interactions with Ala90, Tyr716, Tyr718, and in pi-sigma interaction with Tyr715 from domain III. The N-glycan at Asn720 interacted with Arg710 via H-bonding and Tyr704 via weak van der Waals interactions. A long chain glycan moiety at Asn259 at the outer surface of the enzyme was found to interact via H-bonding with Ser35, Glu36, and Asp229.

Notably, the protein glycans were also shown to exhibit a potential binding affinity for polysaccharides such as cellulose [19] and aromatic compounds [20,21]. This suggests a possible role of N-glycans in promoting cellulose binding and also in protein–glycan interactions. Glycans in β-glucosidases can stabilize the crystal packing contacts and also participate in hydrogen-bonding interactions at the dimer interface [8]. There are two glycosylated Asn residues at the dimer interface of β-glucosidases that provide protein–glycan interactions between the two chains. One of those Asn residues corresponds to Asn531 in *Ct*BGL. This Asn bears only two monosaccharide molecules, whereas, for comparison, the equivalent Asn residue in *Aa*GL1 has seven monosaccharides with the terminal ones able to reach the adjacent subunit. The second Asn residue that participates in dimer interface contacts is not present in *Ct*BGL. The lack of these contacts could therefore contribute to reduced strength of intermolecular interactions in the crystal lattice, resulting in further instability of the *Ct*BGL crystals.

### 2.5. Active Site

**Figure 6:**
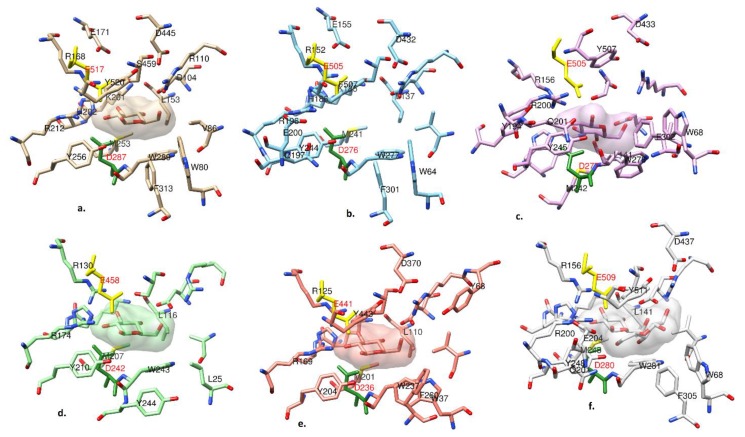
Active site structure comparisons of (**a**) *Ct*BGL, (**b**) *Nc*Cel3A, (**c**) *Re*Cel3A, (**d**) *Tn*Bgl3B, (**e**) *Hj*Cel3A, and (**f**) *Aa*BGL1. The corresponding conserved nucleophile Asp is colored in dark green and the acid/base Glu is colored in yellow. Both residues are labeled in red. Carbon atoms are colored differently in each enzyme. The active site BGC is displayed with its solvent-excluded surface for visual clarity.

The active site is located in a shallow pocket near the interface of the first and second domain. A molecule of *β*-d-glucose (BGC) was fitted at the active site based on residual electron density observed in electron density *F_o_*–*F_c_* difference maps. The source of the *β*-d-glucose is most likely the growth medium used as no *β*-d-glucose was used during crystallizations or soaking. Two catalytic residues were identified, Asp287 (nucleophile) at the N-terminal TIM-barrel domain and Glu517 (acid/base) at the sandwich α/β domain II. Both catalytic residues were found conserved in the GH3 family members (Figure 6). The corresponding catalytic residues were Asp276/Glu505 in *Nc*Cel3A, Asp277/Glu505 in *Re*Cel3A, Asp280/Glu509 in *Aa*Bgl1, Asp236/Glu441 in *Hj*Cel3A, and Asp242/Glu458 in *Tn*Bgl3B. The collapsed TIM-barrel model of domain I is vital for the proper accession of the active site. It was found that near the active site, the second barrel β strand (Gly87 to Thr89) was much shorter and antiparallel, which creates an active site much wider and accessible when compared with GH3 enzyme structures with complete TIM-barrel fold [6]. An additional electron density present at the active site next to BGC was not interpretable and may suggest a bound buffer molecule, MPD from the crystallization mother liquor, or a partially bound glucose molecule.

### 2.6. CtBGL Thermostability

*Ct*BGL has been found to be thermostable at 50 °C and to retain half of its activity after incubation at 65 °C for 55 min [22]. The enzyme also retains 29.7% of its activity after incubation at 70 °C for 10 min. In regard to most other β-glucosidases from thermophilic fungi, *Ct*BGL exhibits comparable thermostability, as previously reported [22]. In contrast, *A. fumigatus* β-glucosidase has been found highly thermostable and able to retain most of its activity for at least 19 h at 65 °C [23]. 

Protein thermostability is usually hard to predict and there is no a common mechanism yet available [24,25]. Several factors of protein thermostability have been proposed that could provide some clues (Table 4). Solvent-accessible surface (SAS), charged residues, and glycosylation patterns are some of the key indicators. The structure of *Hj*Cel3A from the mesophilic fungus *Hypocrea jecorina* had the lowest SAS (22812 Å^2^), owing to the smaller number of residues and the lack of loop V. Similarly, loop V was absent in *Tn*Bgl3A and the SAS value was reduced. The SAS values for the other β-glucosidases, including *Ct*BGL, were quite similar, i.e., around 28100 Å^2^.

Charged residues in the structures can contribute to structural integrity and, in turn, to thermostability. The negatively (Asp and Glu) and positively (Arg and Lys) charged residues may provide a stability profile of the structure [26]. The numbers showed significant variations when the proteins were compared. In particular, *Hj*CelA showed reduced numbers of positively and negatively charged residues, despite its high thermostability with an optimum temperature at 90 °C and an unfolding temperature of ~88 °C depending on the enzyme concentration [27]. The number was increased for the β-glucosidases from other thermophilic and mesophilic β-glucosidases. In addition, *Afβ*G, despite its high thermostability, was also characterized by a similar content of charged residues, suggesting that the strength of individual ion–pair interactions may play a key role.

Finally, the glycosylation pattern found may also contribute to the thermostability of the enzyme by promoting interactions with amino acid residues. It has been shown that glycosylation enhances the solubility, reduces the aggregation, and increases the thermal stability of proteins [28,29]. The exact mechanism by which the enzyme glycosylation pattern affects the overall function and structure of proteins is not yet well understood. Analysis of protein structures deposited in the Protein Data Bank has suggested that N-glycosylation causes no significant local or global structural changes; however, it decreases the protein dynamics, thus leading to increased stability [26]. Aglycosylated proteins are, in general, found to be less stable, and therefore aggregate more easily than its glycosylated counterparts at certain temperatures. Thus, glycosylation has been suggested as a potential factor to enhance enzyme solubility, stability, and function [30]. Thermostability measurements in *Hj*Cel3A samples with different degree of N-glycosylation revealed the same melting temperature (74.0 ± 0.2 °C), thus suggesting that the effect of glycosylation may be case-specific and other factors could play a role. Interestingly, *Tn*Bgl3B that lacks glycosylation exhibited thermostability, most likely as a result of the high numbers of charged residues. Nevertheless, the moderate glycosylation in *Ct*BGL could not be ruled out as a contributing factor to the limited stability of the enzyme compared to other more thermostable β-glucosidases characterized by extensive glycosylation. Further studies are, however, required to better understand the role of glycosylation in β-glucosidases and in protein stability.

## 3. Materials and Methods 

### 3.1. Protein Expression, Purification, and Crystallization

Protein expression and purification of the β-glucosidase from *Chaetomium thermophilum* CT2 was carried out as previously described [22]. Briefly, the enzyme (Uniprot id A6YRT4) was produced in *Pichia pastoris* GS115 cells and purified by ion-exchange chromatography on a DEAE-Sepharose (Pharmacia, Uppsala, Sweden) to homogeneity as judged by SDS-PAGE. β-Glucosidase activity was assayed with salicin using the Miller’s method and also detected in native polyacrylamide gel using 4-methylumbeliferyl-β-d-glucopyranoside [22]. The activity of the enzyme was 1.62 ± 0.20 U/mg (1 U corresponds to the release of 1 µM of glucose per min) from three independent measurements at optimum conditions of pH and temperature.

### 3.2. Protein Crystallization

Prior to crystallization, the enzyme solution was concentrated to ~10 mg/mL with Amicon^®^ Ultra Centrifugal Filters (10,000 MW cut-off) (Millipore, MA, USA) in 10 mM HEPES–NaOH, pH 7.0 buffer. Crystals were obtained by the hanging-drop vapor diffusion method at 16 °C using a well solution of 35–45% *v*/*v* MPD (Sigma-Aldrich, St. Louis, MO, USA). The drops were prepared by mixing 2 µL of protein solution with an equal volume of well solution. The crystals grew as octahedra to a maximum size of approximately 0.06 × 0.06 × 0.08 mm^3^ within a period of 1 month.

### 3.3. Data Collection and Processing

Data were collected on the X13 beamline at EMBL-Hamburg (c/o DESY) from a single crystal under cryogenic (100 K) temperature using a MARCCD detector. The presence of MPD in the crystallization was sufficient for cryoprotection, thus no additional cryoprotectant was needed. One hundred and fifty diffraction images were collected from a single crystal with a rotation range of 0.45° per image. Data processing was carried out with XDS [32]. The crystal was found to belong to the tetragonal space group *P*4_1_2_1_2/*P*4_3_2_1_2. Assuming one molecule in the asymmetric unit, the Matthews coefficient V_M_ [33] is 5.4 Å^3^/Da corresponding to a solvent content of ~77%.

### 3.4. Structure Determination and Refinement

Initial phases were obtained with molecular replacement using Phaser [34], as implemented in Phenix 1.15.2_3472 [35]. The crystal structure of *Aspergillus aculeatus* β-glucosidase in complex with castanospermine (PDB id 4iif; sequence identity 61.5%) was used as a search model after pruning side chains with Sculptor [36] based on sequence alignment considerations. Initially, the search was carried out assuming two molecules in the asymmetric unit, but no solution was produced. Based, however, on the statistics for one molecule (TFZ = 30.9), the search was limited to one molecule and a single solution was obtained in space group *P*4_1_2_1_2. Refinement was carried out using simulated annealing (1000 K) in Phenix with maximum likelihood as target function. The refinement was alternated with model visualization and rebuilding using Coot 0.8.9 [37]. Tight restraints were used to avoid overfitting of the structure to the data, owing to the low resolution. The progress of the refinement was monitored by the *R*_free_ with 5% of the reflections used for the calculations [38]. High-resolution structures were used to assist the rebuilding in places with poor electron density and ambiguities in atom positions. Structure validation was performed with tools implemented in Phenix and Coot. The stereochemistry and conformation of the sugars were tested with Privateer [39]. Data collection and final refinement statistics are shown in Table 1. The atomic coordinates and the structure factors have been deposited to the Protein Data Bank under the accession code 6SZ6.

### 3.5. Structure Analysis

PDBeFold [40] was employed to identify structural similarities between *Ct*BGL and deposited PDB structures. Structure-based sequence alignment was performed with UCSF Chimera 1.13.1 [41]. ESPript 3 [16] was used in rendering sequence similarities and secondary structure information from aligned sequences. Salt bridges were calculated using ESBRI (http://bioinformatica.isa.cnr.it/ESBRI/). SAS area was calculated using PDBePISA [42]. The values for charged residues were calculated using the ExPASy server (http://web.expasy.org/protparam/) ProtParam tool [43].

## 4. Conclusions

The *Ct*BGL structure was determined at 2.99 Å resolution and refined to good stereochemical and refinement numbers, despite the limited resolution. The structure exhibited a three-domain architecture with two linker regions as other β-glucosidase structures. Variations in the length of the first linker regions and the third domain were identified. *Ct*BGL showed the highest structural similarity with *Nc*Cel3A. The catalytic residues at the active site were identified as Asp287 (nucleophile) and Glu517 (acid/base). *Ct*BGL showed a low number of glycans at glycosylation sites compared to other heavily glycosylated GH3 β-glucosidases. Charged residues and the glycosylation pattern are suggested as potential contributing factors for the thermostability properties of *Ct*BGL. The analysis presented in this study could offer new ideas towards further improvements in β-glucosidases for better use in biotechnological applications.

## Figures and Tables

**Table 1:** Data collection and refinement statistics.

**Data Collection**	
Beamline	EMBL-DESY X13
Wavelength (Å)	0.8123
**Data Processing**	
Space group	*P*4_1_2_1_2
Unit cell dimensions a, b, c (Å)	121.9, 121.9, 264.9
No. of molecules/asymmetric units	1
Mosaicity (°)	0.070
Resolution range (Å)	20.0–2.99 (3.11–2.99) *
Total measurements/unique reflections	222508 (22974)/41051 (4513)
Completeness (%)	99.3 (98.6)
<I/σ(I)>	7.8 (1.4)
*R*_meas_	0.287 (1.452)
*R*_pim_	0.122 (0.846)
CC_1/2_	0.978 (0.492)
Wilson B-factor (Å^2^)	46.2
**Refinement**	
Reflections (working/test)	38861/2047
*R*_cryst_/*R*_free_	0.201/0.249
No. of protein atoms	6457
Ligand atoms	12
No. of Sugar Atoms	355
No. of Water Molecules	6
**Geometry**	
rmsd in bond lengths (Å)	0.007
rmsd in bond angles (°)	0.94
Most favorable regions (%)	92.8
Additional allowed regions (%)	6.2
Outliers (%)	1.0
Clashscore	10.4
**Average B-Factors (Å^2^)**	
Protein	50.4
Ligand	63.8
Sugars	72.4
Water molecules	32.8
**Pyranose Conformations (Total/Percentage)**	
Lowest energy conformations	23/85.2
Highest energy conformations	4/14.8

* Values for the outermost resolution shell are shown in parentheses.

**Table 2:** Glycosylation sites and glycan descriptions in *Ct*BGL.

Residue	Corresponding Glycan Structure
Asn72	Man-*β*1,4–GlcNAc-*β*1,4–GlcNAc
Asn259	Man-*β*1,4–Man-*β*1,4–GlcNAc-*β*1,4–GlcNAc
Asn322	GlcNAc-*β*1,4–GlcNAc
Asn329	Man-*α*1,2–Man-*α*1,6*–(α*1,3-Man)-*α*1,6–Man-(*α*1,3-Man)Man-*β*1,4–GlcNAc-*β*1,4–GlcNAc
Asn504	GlcNAc
Asn531	Man-*β*1,3–GlcNAc
Asn572	GlcNAc-*β*1,4–GlcNAc
Asn698	GlcNAc
Asn720	Man-*β*1,4–GlcNAc-*β*1,4–GlcNAc
Asn744	GlcNAc

**Table 3:** Comparison of glycosylation sites ^%^.

*Ct*BGL	*Nc*Cel3A(5nbs)	*Aa*Bgl1 (4iif)	*Re*Cel3A (5ju6)	*Hj*Cel3A (3zyz) ^#^
Asn72 (+)	Asn57 (+)	Asn61 (+)	Asn61 (+)	Gly30 (−)
Asn259 (+)	Asn248 (+)	Asn252 (+)	Asn249 (+)	Asn208 (+)
Asn322 (+)	Asn311 (+)	Asn315 (+)	Asn312 (+)	Ala270 (−)
Asn329 (+)	Asn318 (+)	Asn322 (+)	Asn319 (+)	Ser277 (−)
Asn504 (+)	Asp492 (−)	Ala496 (−)	Ala492(−)	Lys428 (−)
Asn531 (+)	Asn519 (+)	Asn523 (+)	Asn519 (+)	Asn455 (−)
Asn572 (+)	Asn560 (+)	Asn564 (+)	Asn560 (+)	Gln496 (−)
Asn698 (+)	Ser687 (−)	Asn690 (+)	Asn685 (+)	
Asn720 (+)	Asn710 (+)	Asn712 (+)	Asn707 (+)	
Asn744(+)	Lys734 (−)	Gly736 (−)	Asn731 (+)	

^%^ (+) depicts glycosylation, (−) absence of glycosylation ^#^ In subunit A.

**Table 4:** Comparison of thermostability parameters.

	*CtBGL*	*Afβ*G (5FJI)	*Aa*Bgl1 (4IIF)	*Re*Cel3A (5JU6)	*Tn*Bgl3B (2X40)	*Hj*Cel3A (3ZYZ)
Thermal stability characteristics	*T*_1/2_ 65 °C after 55 min	19 h at 65 °C [23]	*T*_1/2_ 62 °C after 30 min [31]	*T*_m_ 87.3 °C [4]	Unfolding temperature 86–89 °C [27]	*T*_m_ 77.6 °C [4]
Asp + Glu (−)	93	95	92	87	113	55
Arg + Lys (+)	66	67	65	59	93	51
Pro: Gly	0.51	0.55	0.51	0.61	0.58	0.52
Val (%)	7.0	7.9	8.0	7.5	9.2	8.8
Amino acid residues	867	844	841	857	721	714
SAS (Å^2^)	28,502.9	28,092.1	28,074.9	28,481.7	26,430.0	22,551.0
Intra-chain salt Bridges	46	49	42	44	39	34
H-bonding interactions	12 ^∞^	22	25	22	23	8
Interface area, Å^2^	1544.2 ^∞^	1324.3	1445.3	1322.5	1253.1	699.3
Helical content (%)	21.2	21.8	21.8	20.5	26.5	23.7
Sugars	27	45 (chain A)	44 ^%^	50 (chain C)	NA ^$^	2

^∞^ In the crystallographic dimer. ^%^ Prior to crystallization, the enzyme was subjected to endoglycosidase H treatment with ~50% of the N-glycans removed [3]. ^$^ The enzyme was produced in *Escherichia coli.*

## Abbreviations

BGC	β-d-glucose
GH3	Glycoside hydrolase family 3
MPD	2-methyl-2,4-pentanediol
PDB	Protein Data Bank
NCS	Non-crystallographic symmetry
rmsd	Root mean square deviation
SAS	Solvent-accessible surface
*T*_1/2_	Half-life inactivation
*T*_m_	Melting temperature
